# Awareness Among Educated and Uneducated Parents of Beta-Thalassemia Major Patients About Antenatal Screening

**DOI:** 10.7759/cureus.28561

**Published:** 2022-08-29

**Authors:** Lal Muhammad, Khawaja Kamran Wajid, Inayatullah Afridi, Sami Ullah, Afzal Khan, Amir Muhammad

**Affiliations:** 1 Pediatrics, Lady Reading Hospital Medical Teaching Institution, Peshawar, PAK; 2 Pediatric Medicine, Lady Reading Hospital Medical Teaching Institution, Peshawar, PAK

**Keywords:** β-thalassemia, beta thalassemia major, education of the parents, parental awareness, antenatal screening

## Abstract

Objective: This study aims to determine the awareness among educated and uneducated parents of beta-thalassemia major patients about antenatal screening.

Setting: The study was conducted by the Lady Reading Hospital Medical Teaching Institution (MTI), Peshawar.

Duration of the study: This study was conducted for six months.

Materials and methods: A total of 296 parents of beta-thalassemia major were interviewed. Parents who were not ready to give consent and other hemolytic anemia patients were excluded. Data were collected on self-structure pretested Performa. The demography of the patients and awareness of the parents regarding antenatal screening were recorded from each participant. A chi-squared test was used to assess the association between parental education and antenatal screening. A p-value of less than 0.05 was considered significant.

Results: A total of 296 patients were studied; 160 were males (54.1%), and 136 were females (45.9%). The mean age was 6.84 years. Mothers (74.3%) accompanied majority of the patients. Most of the parents (265; 89.5%) were uneducated, and 80% of the parents were not aware of any antenatal screening.

Conclusion: There was a significant association between the education of parents and awareness of antenatal screening (p = 0.01).

## Introduction

Thalassemia is a hereditary disorder in which there is inadequate globin chain production, which in turn leads to ineffective erythropoiesis and anemia [1].

In beta-thalassemia, the synthesis of the beta chain of hemoglobin is decreased, which results in varying phenotypes ranging from severe anemia to clinically normal individuals. There are three main types of thalassemia: thalassemia major, thalassemia intermedia, and thalassemia minor. Thalassemia major is the most severe form, which presents in infancy and requires frequent red blood cell (RBC) transfusions [2].

Beta-thalassemia is highly prevalent in the Middle East, India, Pakistan, and South East Asia. Approximately, 9,000 children with beta-thalassemia are born every year in Pakistan. The estimated carrier rate is 5%-7%, which accounts for 9.8 million carriers in the total population. The average life expectancy of thalassemic patients in Pakistan is 10 years [1].

In Pakistan, the current prevalence of the gene for β-thalassemia is 5­%-8%, which makes up for almost 5% of the total cases in the world [3-8]. The numbers are increasing because of the lack of awareness and insufficient education campaigns [4]. It has been observed that the majority of the mothers with thalassemia trait do not know about their carrier status and as a result give birth to a thalassemia major child [6-10].

In a multicenter study, 26% of parents of beta-thalassemia major were not aware of prenatal screening in thalassemia, while 61% of total parents of the study were illiterate, but no parents have done prenatal screening, and only 10 parents had done premarital screening [11]. In our society, consanguineous marriages are common without premarital screening or counseling of a family having a history of thalassemia. Antenatal screening is not readily available, and termination of pregnancy is an ethical and religious issue. So, thalassemia is managed with blood transfusions, which leads to complications and ends in death, while bone marrow transplantation is not possible for a such large group of patients with beta-thalassemia major. Abortion during pregnancy is allowed in case of endangering to mother's life and fetal anomalies in Islam before 120 days of pregnancy [12].

In Saudi Arabia, the majority of the parents of hemoglobinopathy patients accepted prenatal diagnosis (81.3%) [13], but it was found that 8%-12% of the parents did not like the termination of pregnancy for any of the 30 inherited conditions [14]. The only path to stop the disease and decrease its complications is by giving knowledge to the general population about it [1].

For this reason in this study, awareness among educated and uneducated parents of thalassemia major patients about antenatal screening is designed.

## Materials and methods

Objective

The objective of the study was to determine awareness among educated and uneducated parents of beta-thalassemia major patients about antenatal screening.

Operational definition

Antenatal Screening

Antenatal screening for beta-thalassemia major was done by chorionic villus sampling in the first trimester of pregnancy.

Unaware Parents

Those who do not know about antenatal screening are called unaware parents.

Partial Awareness

They have some knowledge of antenatal screening.

Full Awareness

They have full awareness about antenatal screening like the timing of the test, procedure, and laboratory name.

Educated Parents

These parents have matric (secondary school certificate/10 years of basic education) or higher education.

Uneducated Parents

These parents have under matric education.

Socioeconomic Status

If the income was less than 30,000 PKR (Pakistani rupees), the socioeconomic status was considered poor. If the income was more than 30,000 PKR, the socioeconomic status was considered good.

Materials and methods

Setting

The study was conducted in the Department of Pediatrics, Medical Teaching Institute, Lady Reading Hospital, Peshawar.

Sample Size

The sample size was 296, using 26% of unaware parents of beta-thalassemia patients, 95% confidence level, and 5% margin of error, under WHO software for sample size determination.

Sampling Technique

A consecutive (non-probability) sampling technique was used.

Duration of Study

The duration of the study was a period of six months minimum from the date of approval of this protocol.

Study Design

This is a descriptive cross-sectional study.

Inclusion criteria

 All parents of patients having beta-thalassemia major were admitted for blood transfusion.

Exclusion criteria

Parents of the patients with all other hemolytic/non-hemolytic anemia and parents of the patients with beta-thalassemia major who were not ready to give consent were excluded from the study. These confounders, if included in the study, will lead to bias in the study results.

Data collection procedure

Approval was taken from the ethical committee of the hospital. Patients meeting inclusion criteria having HbF ≥ 70% and admitted for blood transfusion were included in the study. An informed written consent, mentioning that this information will be used for research purposes and their secrets will be kept confidential, was taken from the parents. A brief history including name, age, gender of patients, and education of parents accompanying the patients was taken from the parents. Under predesigned Performa, awareness of parents about antenatal screening for beta-thalassemia was asked. The confounding factors and bias were controlled by strictly following the exclusion criteria.

Data analysis

All the results collected will be entered into the statistical SPSS (Statistical Package for the Social Sciences) software, version 20 (IBM Corp., Armonk, NY).

## Results

Despite limited resources, data from 296 patients were collected. The total number of male patients was 160 (54.1%) and female patients was 136 (45.9%). The minimum age of the patient was one year, while the maximum age was 17 years with a mean of 6.84 years. A total of 220 patients were accompanied by mothers (74.3%), 44 patients were accompanied by fathers, and the rest by both. The majority of the parents (265, 89.5%) were uneducated, i.e., under matric; 26 parents were educated, i.e., matric and above, while five were highly educated. The majority of parents (237, 80.1%) were not aware of antenatal screening, only 30 parents were fully aware, while 29 were partially aware (Table 1).

**Table 1:** Demographical characteristics of beta-thalassemia major patients

Age of the patient	Minimum	Maximum	Mean
	1 year	17 years	6.8 years
			Percentage
Gender of patient	Male	160	54.1%
	Female	136	49.9%
Accompanying parent	Mother	220	74.3%
	Father	44	14.9%
	Both	32	10.8%
Socioeconomic status	Poor	260	87.88%
	Good	36	12.16%
Education of the parents	Uneducated	265	89.5%
	Educated	26	8.8%
	Highly educated	5	1.7%
Awareness of the parents	No awareness	237	80.1%
	Partial awareness	29	9.8%
	Complete awareness	30	10.1%

The relationship between education of parents and awareness about antenatal screening was found significant using the chi-squared test with a p-value of 0.01 (Table 2).

**Table 2:** Education of the parents compared with awareness of antenatal screening

Awareness of Parents on Antenatal Screening				
Education of the parents	No awareness of antenatal screening	Partial awareness about antenatal screening	Complete awareness about antenatal screening	Chi-square test
Uneducated (under matric)	218	25	22	0.01
Educated (matric)	17	4	5	
Highly educated	2	0	3	

## Discussion

Our study result showed a significant association between the education of the parents of beta-thalassemia major patients and the awareness of antenatal screening. A majority of the unaware parents (80.1%) were uneducated. A study done by Ghafoor et al. showed that 60% of parents do not have much knowledge about thalassemia and 69% were uneducated [15]. Most of the mothers were uneducated (89.5%), which is the main reason for the lack of knowledge about antenatal screening for beta-thalassemia major. In our study, the illiteracy rate is higher than the study done in Karachi 57% and 67% in Turkey [1,16]. Most of the patients belong to a poor family background in our study. Thalassemia is brought under control in a very good manner in Iran, Greece, and Italy by educating the parents and giving them awareness about the disease [1].

In our study, only 16 patients out of 162 having family history of thalassemia major were aware of antenatal screening. So, awareness of families having beta-thalassemia major is poor. It is similar to a study conducted in Iran where 78.6% of parents did not screen themselves in spite of a positive family history of beta-thalassemia major [17]. But in developed countries with good legislation, the effectiveness of screening was improved from 30% to 86% [18]. 

Developed countries focus more on prevention through identifying thalassemia carriers and premarital counseling. In Sardinia, the incidence has decreased from 1:250 to 1:1000 live births. A cross-section of new couples and first-trimester pregnancies were taken. Awareness was created among people through mass media or teaching season. It is also included in the educational syllabus [19], so such strategies are required in Pakistan.

The limitation of our study is that it was a single-center study. As most of the patients came to the public hospital, most of them belong to poor socioeconomic status. So the study should be done in a multicenter/community with the involvement of people of all socioeconomic statuses.

## Conclusions

Beta-thalassemia major is a genetically inherited debilitated disease. It can be prevented by awareness of the parents regarding antenatal screening. Our results showed a significant association between the education of the parents and the awareness of antenatal screening.
